# Tachycardia–bradycardia syndrome

**DOI:** 10.1093/ehjcr/ytag197

**Published:** 2026-03-12

**Authors:** Ranjan Kumar Singh

**Affiliations:** Practicing Physician, Ex. Medical Superintendent, JPN Hospital, Gaya-823001, Bihar, India

## Case description

A 40-year-old woman presented with persistent shortness of breath New York Heart Association (NYHA Class II) for 15 days and was experiencing fatigue for several years. She had no history of rheumatic fever, thyroid disorder, or receiving chronotropic drugs.

**Figure 1:**
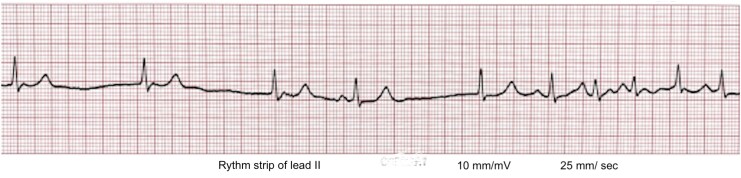
ECG rhythm strip of lead II showing AV node rhythm along with multifocal atrial tachycardia.

On clinical examination, there was no peripheral oedema; Jugular Venous Pressure (JVP) was not raised, but pulse was irregular at 48 beats per minute. Auscultation revealed a loud pulmonary sound (P2) and a Grade 3/4 harsh mid-diastolic murmur in the mitral area. The blood tests were unremarkable, with haemoglobin 11.5 g/dL (reference: 11.5–15), serum creatinine 0.8 mg/dL (reference: 0.7–1.2), and serum potassium and sodium of 4.6 mEq/L (reference: 3.5–5.5) and 138 mEq/L (reference: 135–145), respectively. A chest X-ray revealed cardiomegaly with a cardiothoracic ratio of 0.7, a double density over the right border, and enlargement of the left atrial appendage causing straightening of the left border—suggestive of mitral stenosis (see Supplementary material online, *Figure S1*). An Electrocardiogram (ECG) rhythm trace (*Figure 1*) showed sinoatrial (SA) node dysfunction with atrioventricular (AV) nodal escape rhythm at 42 per minute and bursts of multifocal atrial tachycardia (MAT). Nodal escape rhythm shows retrograde *P*-wave after QRS complex, while varying *P*-wave morphology, varying PR interval, and irregular ventricular rate > 100/min, indicative of MAT.^1^ Although atrial fibrillation (AF) is a common arrhythmia in patients with mitral stenosis, left atrial enlargement may trigger arrhythmias other than AF. Presence of multifocal atrial tachycardia and AV nodal escape rhythm suggests tachycardia–bradycardia syndrome, which occurs in half of cases of SA node dysfunction.^2^ Sinoatrial node dysfunction can occur at any age but is common in elderly persons, and it results from intrinsic causes that include fibrosis, ion channel dysfunction, and remodelling of the SA node.^3^ This case underscores the importance of recognizing complex arrhythmias beyond AF in the setting of mitral valve disease, as the management of tachycardia–bradycardia syndrome requires a nuanced approach. The patient was referred to a cardiac centre.

## Supplementary Material

ytag197_Supplementary_Data

## Data Availability

The data underlying this article are available in the article and its Supplementary material.
