# Supplementary figures and images for: Tachycardia–bradycardia syndrome
Source: Eur Heart J Case Rep. 2026 Mar 12;10(4):ytag197. doi: 10.1093/ehjcr/ytag197 (PMC13064509; doi:10.1093/ehjcr/ytag197)

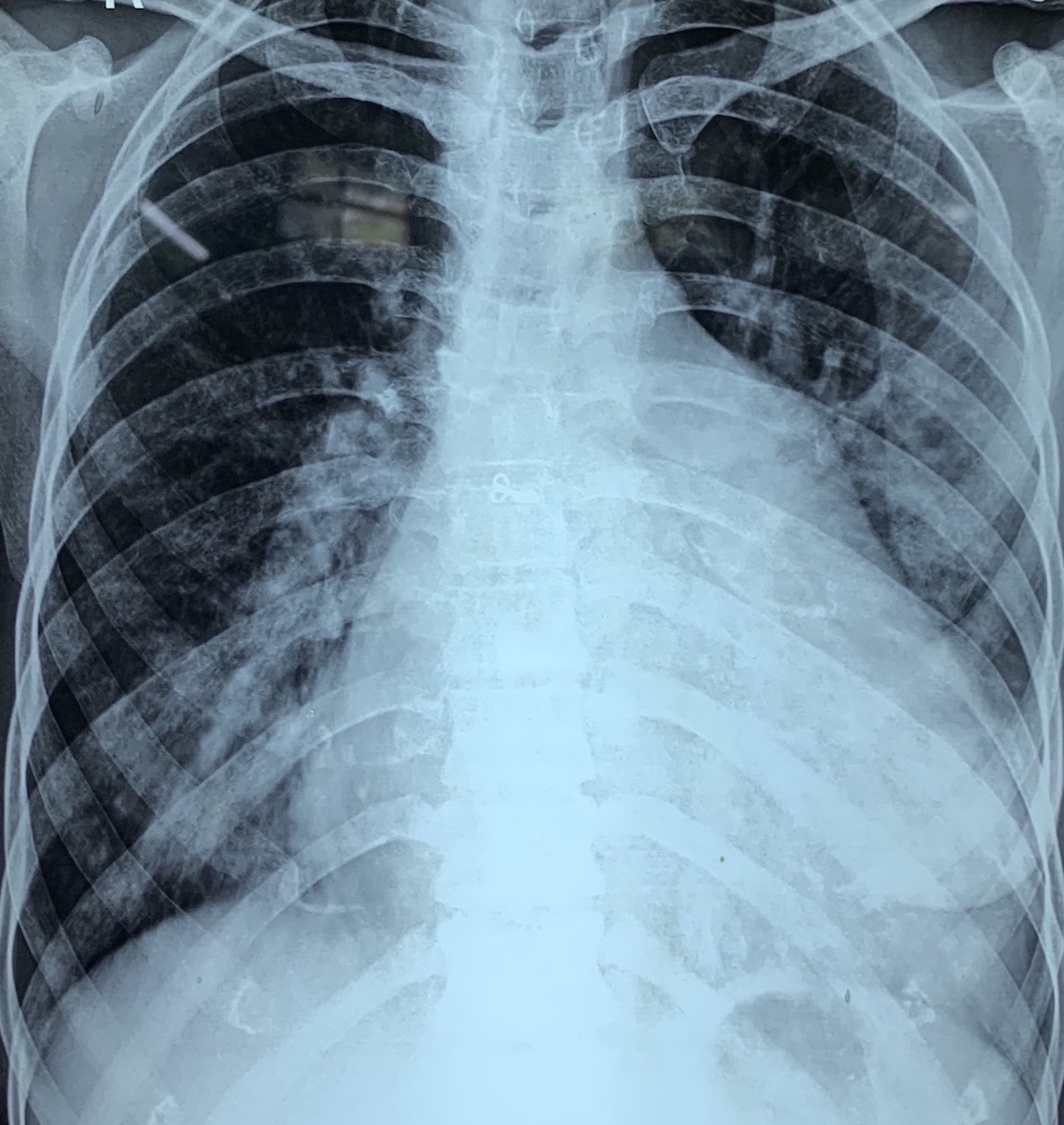

Supplement: ytag197_Supplementary_Data [file ytag197_supplementary_data.zip › CXR_SA node_dysfunction.jpg]
